# Sitting Tai Chi Improves the Balance Control and Muscle Strength of Community-Dwelling Persons with Spinal Cord Injuries: A Pilot Study

**DOI:** 10.1155/2015/523852

**Published:** 2015-01-21

**Authors:** William W. N. Tsang, Kelly L. Gao, K. M. Chan, Sheila Purves, Duncan J. Macfarlane, Shirley S. M. Fong

**Affiliations:** ^1^Department of Rehabilitation Sciences, The Hong Kong Polytechnic University, Hung Hom, Hong Kong; ^2^Department of Orthopaedics and Traumatology, The Chinese University of Hong Kong, Shatin, Hong Kong; ^3^Institute of Human Performance, The University of Hong Kong, Pokfulam, Hong Kong

## Abstract

*Objective*. To investigate the effects of sitting Tai Chi on muscle strength, balance control, and quality of life (QOL) among survivors with spinal cord injuries (SCI). *Methods*. Eleven SCI survivors participated in the sitting Tai Chi training (90 minutes/session, 2 times/week for 12 weeks) and eight SCI survivors acted as controls. Dynamic sitting balance was evaluated using limits of stability test and a sequential weight shifting test in sitting. Handgrip strength was also tested using a hand-held dynamometer. QOL was measured using the World Health Organization's Quality of Life Scale. *Results*. Tai Chi practitioners achieved significant improvements in their reaction time (*P* = 0.042); maximum excursion (*P* = 0.016); and directional control (*P* = 0.025) in the limits of stability test after training. In the sequential weight shifting test, they significantly improved their total time to sequentially hit the 12 targets (*P* = 0.035). Significant improvement in handgrip strength was also found among the Tai Chi practitioners (*P* = 0.049). However, no significant within and between-group differences were found in the QOL outcomes (*P* > 0.05). *Conclusions*. Twelve weeks of sitting Tai Chi training could improve the dynamic sitting balance and handgrip strength, but not QOL, of the SCI survivors.

## 1. Introduction

Good sitting balance control is essential for people with spinal cord injury (SCI) because they are often confined to a sitting position when performing activities of daily living (ADL). The ability to shift their weight voluntarily in different directions without losing stability is important. Sitting balance heavily influences wheelchair skills, especially for performing the wheelies required to ascend or descend a curb or other obstacles. Sitting posture and balance also directly affect transfer performance [1] where the ability to precisely and accurately control intentional movements of the center of pressure (COP) in different directions is important. Better directional control provides more accurate controlling of a variety of transfer activities. In a recent survey, thirty-one percent of 659 community-dwelling wheelchair users with SCI reported a total of 553 fall events [2]. The major factor contributing to falls is often a loss of balance during transfers [3].

Wheelchair users with SCI have decreased or no control of their trunk, leading to poor sitting balance and stability which may cause falls during transfer [4]. With advances in rehabilitation strategies, people with SCI can benefit from various interventions that restore their functional capacity and ability to reenter the community, but little has been published describing exercise programs to improve the sitting balance control of people with SCI who live in the community.

Good quality of life (QOL) is related to good relationships, maximizing functioning, engaging in activities, and the ability to access one's environment [5]. There are several factors influencing QOL in people with SCI. Functional losses have a great impact on QOL. Ville and Ravaud [6] pointed out that the functional independence is directly linked to QOL. In other words, the impact of limited functional status on social activity could reduce QOL. Participation in the community has a direct effect on QOL through increased opportunities for meaningful activity and social connections. After discharge from the hospital, regular exercise is not readily available. A new approach is needed to improve the functional status and QOL of community-dwelling people with SCI. Tai Chi is a Chinese martial art which is regarded as a gentle, relaxing, yet invigorating form of exercise. Tai Chi is both an integrated exercise and an enjoyable sport for all kinds of people: strong and weak, young and old, and male and female. Weather does not inhibit its practice, as it can be performed indoors. Traveling time and space requirements are minimal [7].

Previous academic work has shown that Tai Chi practice improves standing balance control, muscle strength, functional status, aerobic capacity, and arterial compliance and decreases fall risk in ambulatory older population [8–13]. Using the limits of stability test, Tsang and Hui-Chan [14] showed that Tai Chi practitioners could initiate voluntary shifting of their weight to different spatial positions within their base of support more quickly than control participants, leaned further without losing stability, and showed better control of their leaning trajectory in standing. However, the traditional Tai Chi forms pose difficulty for older adults with poor standing balance or who are physically dependent and may increase the risk of falling or injuries. In view of this, different Tai Chi modifications have been proposed for the frail elderly or individuals with physical disabilities [15–17]. Based on the existing evidence, we postulated that practising Tai Chi in sitting might also benefit the nonambulatory individuals, both physically and psychologically. To date, no study has investigated the effect of sitting Tai Chi on physical and psychological outcomes in the wheelchair-bound individuals.

Community-dwelling people with SCI lack an effective exercise to improve their sitting balance. In light of this clinical need, our research team has designed a 12-form sitting Tai Chi routine specifically for people with SCI based on the traditional forms of Yang's style Tai Chi. In this sitting Tai Chi form, shifting of weight while sitting, trunk rotation, upper limb joint mobilization, and muscle strengthening were emphasized in an attempt to counteract loss of strength in the lower limb and trunk muscles and unstable sitting balance. The training was safe and simple for the participants to perform sitting in a wheelchair or chair [18]. In addition, this sitting Tai Chi form could be performed easily without any time and venue constraints. However, whether sitting Tai Chi training could improve muscle strength, sitting balance, and QOL in people with SCI was still unknown. This study aimed to explore the effects of sitting Tai Chi on these outcome parameters in people with SCI.

## 2. Methods

### 2.1. Study Design and Participants

Nineteen wheelchair users with SCI (11 males and 8 females aged 47.9 ± 10.7 years) participated in either Tai Chi intervention or control group activities according to their preferences. The participants were recruited from the Hong Kong Sports Association for the Physically Disabled, the Direction Association for the Handicapped, and the Paraplegic & Quadriplegic Association. They were given both written and verbal information about the study before giving their informed consent to participation. The inclusion criteria were at least 1 year after injury, incomplete injury according to the American Spinal Injury Association (ASIA) Impairment Scale (i.e., ASIA B, C, and D) [19], aging 18 years or above, and being able to communicate and follow instructions. People with unstable cardiopulmonary disease, serious complications related to the SCI (e.g., pressure ulcers), contracture or marked muscle hypertonicity, poorly controlled hypertension, or metastatic cancer were excluded. The study protocol was approved by the Ethics Committee of the Hong Kong Polytechnic University and written informed consent was obtained from all participants before the study started.

### 2.2. The Tai Chi Intervention

The sitting Tai Chi intervention involved two 90-minute sessions, 2 times per week for 12 weeks. A 12-form sitting version of Yang's Tai Chi style was designed for the experiment. Details of this sitting Tai Chi form were reported in our previous study [18]. A Tai Chi master and a physiotherapist conducted the training. Each session included a 5-minute warm-up and 5-minute cool-down with rests as necessary. The whole 12 forms required approximately 3 minutes to complete and it took 6 lessons for the participants to learn the whole sitting Tai Chi routine (2 forms for each lesson). The participants spent the rest of the training sessions in polishing the forms and engaging in sitting balance training. Attendance was recorded. The participants were encouraged to also practice at home for 90 minutes each week. A logbook was used to monitor home exercise by self-recording.

Participants in the control group were involved in educational talks and social activities of equivalent duration and frequency. Baseline and post-intervention measurements of sitting balance control, handgrip strength, and QOL were conducted for both groups.

### 2.3. Outcome Measurements

#### 2.3.1. Muscle (Handgrip) Strength

Handgrip strength has been regarded as a significant indicator of whole-body strength in the elderly population [20] and is commonly measured to indicate the change in overall muscle strength before and after Tai Chi training [21, 22]. A Jamar hydraulic hand dynamometer (Sammons Preston, Ability One Co., Bolingbrook, IL) was used to measure handgrip strength. The Jamar dynamometer is widely used for measuring isometric grip force from 0 to 200 lb (90 kg) and has shown excellent reproducibility [23, 24]. Participants were instructed to sit in their own wheelchair with the test shoulder adducted and rotated neutrally and the elbow flexed at 90°, the forearm in a neutral position, and the wrist between 0 and 30 degrees of extension and between 0 and 15 degrees of ulnar deviation [24]. Familiarization trials were allowed before the results of three measurements were averaged for comparison.

#### 2.3.2. Sitting Balance Control

The sitting balance tests were performed supported in the participant's own wheelchair. They involved limits of stability test and a sequential weight shifting test. Both were performed on a wooden force platform (90 cm × 90 cm) in front of an adjustable-height screen 1.5 m away on which the COP was continuously displayed. The participant's COP was measured by 4 load cells (SBDEG, Schaevitz Measurement Specialties Inc., VA, USA) under the platform. The measurement range of the load cells was 40–400 pounds of force. All movement data from the force platform were converted and digitized using a Multifunction Data Acquisition device (National Instrument (NI) USB-6009, USA) and an 8-channel analog-to-digital converter at a sampling rate of 1000 Hz. A computer running tailored LabVIEW (version 8.6, National Instruments, USA) software displayed and stored the COP data in real time during the sitting balance tests.

*(1) Limits of Stability*. Supported sitting was defined as seated in the participant's own wheelchair but without resting on the back support for the paraplegics or with both back and head support for the tetraplegics at the beginning of testing. The participant's hips, knees, and ankles were kept at approximately 90° of flexion, and the feet were shoulder width apart resting on the platform. Each participant had practice trials for familiarization, followed by three trials of each balance test.

The limits of stability (LOS) test measured their intentional weight shifting ability in multiple directions within their base of support. The initial COP position was displayed in the center of the screen together with eight target positions in front, right front, right, right back, back, left back, left, and left front. The participants were required to move the COP trace on the screen toward one of the eight target positions by shifting their weight within their limits of stability as quickly and as smoothly as possible when a visual signal indicated a target. There was a 20-second rest period between trials to minimize any fatigue which might affect performance. For each target, the mean value of three trials was calculated for comparison. The investigator was beside the participant for safety. There was a two-second baseline measurement of COP sway before each target was indicated.

The outcome measures of the LOS test were (1) reaction time (RT, in seconds): the time from the appearance of a target to the onset of the voluntary shifting of the COP; (2) maximum excursion (ME, in mm): the maximum displacement of the COP in the target direction; (3) directional control (DC, in %): a comparison of the amount of movement of the COP in the on-target direction with the amount of off-target displacement. This LOS test protocol has been shown in a previous study to have moderate to excellent reproducibility (ICC = 0.751–0.990) [25].

**Figure 1:**
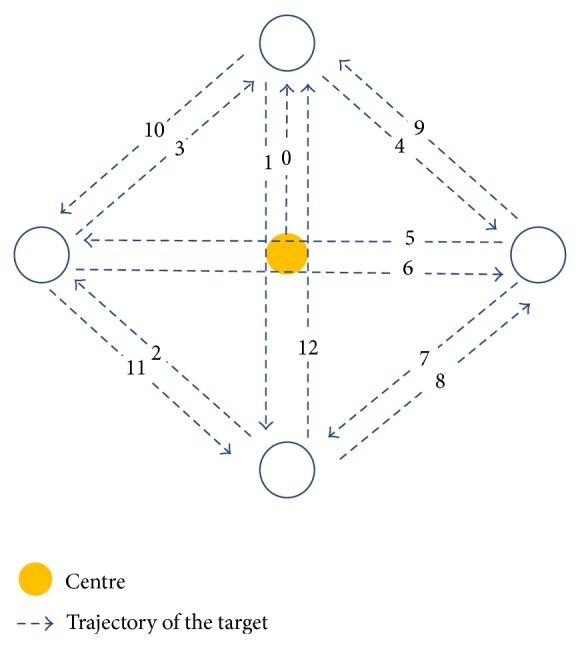
The trajectory of the targets in the SWS test.

*(2) Sequential Weight Shifting*. A temporal-spatial task involving diagonal COP shifting was used to measure sequential weight shifting (SWS) ability. The sitting position was the same as in the LOS test. As soon as a visual target appeared, participants were asked to shift their COP to move the screen trace to the target as quickly as possible without losing their balance. Twelve targets appeared sequentially. When each target was hit, it disappeared and another appeared. The 12 targets appeared above, left, below, and to the right of the center (Figure 1). The distance from the center to each target was 75% of that participant's maximal excursion as determined in the LOS test. The expected (ideal) trajectory is shown in Figure 1. During the test, the participants had continuous visual feedback about the position of their COP from the screen as they performed the weight shifts. The total time for the participant to hit the 12 targets sequentially and directional control were computed. The SWS test has been shown to have moderate to excellent reproducibility (ICC = 0.688–0.952) in a previous study [26].

#### 2.3.3. Quality of Life

The brief version of the World Health Organization's Quality of Life Scale (WHOQOL-BREF) has been demonstrated to be an acceptable and validated questionnaire to assess the QOL of people with SCI [27, 28]. The Chinese version of the WHOQOL-BREF was used to measure QOL in this study. It has previously been shown to have high intrarater reliability (ICC = 0.84–0.98) [29] and its validity as a tool for measuring the QOL of Chinese individuals with SCI has been established [28].

WHOQOL-BREF is a self-reporting questionnaire which consists of 26 questions. There are 2 general questions (about overall QOL and overall health perceptions) and more detailed questions about four QOL domains. Seven query the physical domain (D1), 6 the psychological domain (D2), and 3 the social relationship domain (D3), and 8 questions probe the environmental domain (D4). All questions solicit ratings on a five-point Likert scale. The scores were first summarized for the 4 domains (physical health, psychological health, social relationships, and environment) according to the WHOQOL-BREF Guidelines by taking the mean score for the questions addressing that domain and multiplying by 4. So each domain score could range from 4 to 20. A higher score indicates better QOL [27–29].

### 2.4. Sample Size Estimation and Statistical Analysis

The sample size was determined through power calculation based on the results of a previous study of Tai Chi with older adult participants [29]. Participants in that study achieved significant improvement in balance performance (effect size of 1.11) for the directional control in the limits of stability test measurement after 8 weeks of Tai Chi training when compared with matched controls. Our estimation of sample size was based on repeated measures MANOVA comparing the two groups. With an estimated effect size of 0.80 (lower than the previous effect size because participants in this study received less intensive training and in sitting Tai Chi forms), an overall sample size of 24 participants (12 participants per group) should achieve 80% power at the 0.05 significance level. This power calculation allowed for a 14% dropout rate.

The average age, sitting height, and weight of the experimental and control groups were compared using independent *t*-tests. The sitting height was defined as the vertical distance from vertex of the head to the seat of the wheelchair. Chi-square tests were applied for between-group comparison of the gender distribution and injury levels. After testing for normality and equal variance, independent *t*-tests were employed to analyze any intergroup differences in the baseline values between the Tai Chi group and the control group. If a significant difference was found in any of the baseline values it was treated as a covariate in the subsequent statistical analysis. Two-way repeated measures MANOVA with an intent-to-treat design was employed to analyze any intergroup differences in the outcome measures between before and after the intervention. Multivariate analysis was used to avoid an inflation of type I error due to multiple comparisons. If MANOVA revealed a significant result overall, univariate test was used to identify the significant time-by-group effects. Paired *t*-tests were also conducted to investigate whether there was any within-group difference in the assessment intervals with the baseline values as reference. Independent *t*-tests were conducted to compare the Tai Chi and control groups at the 2 time points. A significance level (*α*) of 0.05 was chosen for the statistical comparisons. The values of effect size of 0.20, 0.50, and 0.80 were used to represent small, moderate, and large effects.

## 3. Results

The demographics of the two groups are summarized in Table 1. There was no significant difference in the gender distribution or in average age, sitting height, weight, spinal cord injury level [30], or time since injury between the two groups. The neurological level of the participants' injuries was determined by a physiotherapist which ranged from C6 to L1 according to the International Standards for the Neurological Classification of Spinal Cord Injury (version 2011). The time since injury averaged 16 years (from 2 to 48 years). Nineteen participants had lesions incomplete to varying degrees (11 participants with ASIA grade B, 3 with grade C, and 5 with grade D impairments).

Eleven participants with SCI participated in the sitting Tai Chi training while 8 participants with SCI participated in the educational talks and social activities as controls. Only one participant in the control group dropped out due to loss of interest (i.e., 87.5% completed). The average attendance of the face-to-face Tai Chi training sessions was 90%, while that of the controls was 87%. No adverse events and complications (e.g., dizziness) were reported during the training period. The self-reported sitting Tai Chi home exercise compliance rate was 100%.

### 3.1. Handgrip Strength

Two-way repeated measures MANOVA test of the bilateral handgrip strength results showed an overall significant time by group interaction (*P* = 0.049; Table 2). Univariate test showed a significant time by group interaction (*P* = 0.019; effect size = 0.285) in terms of the dominant handgrip strength. The effect size of 0.285 was regarded as from small to moderate effect. Paired *t*-tests showed that only the experimental group had a significant improvement in dominant handgrip strength (*P* = 0.008) after three months of training. No significant change was found in the control group (*P* = 0.441). No significant time by group interaction was found involving nondominant handgrip strength (*P* = 0.318); however paired *t*-tests revealed that the Tai Chi group had significant improvement (*P* = 0.027) after three months of training. No significant change was found in the control group (*P* = 0.624).

### 3.2. Sitting Balance Control

The two-way repeated measures ANCOVA of the sitting balance results showed an overall significant time by group interaction (*P* = 0.035; Table 2). Since the average baseline maximum excursions of the two groups were significantly different (*P* < 0.05), this variable was treated as a covariate in the analysis.

#### 3.2.1. Changes in the Limits of Stability

A significant time by group interaction effect (*P* = 0.042; effect size = 0.263) was found in the reaction time. Paired *t*-tests revealed that only the sitting Tai Chi group had significantly better reaction time performance (*P* = 0.025) after three months of training. No significant change was found in the control group over time (*P* = 0.469). Moreover, a significant time by group interaction (*P* = 0.016; effect size = 0.349) was found in the average maximum excursion. Paired *t*-tests revealed that only the sitting Tai Chi group achieved an improvement in the COP distance travelled (*P* = 0.006) after three months of training. No significant change was found in the control group (*P* = 0.613). Furthermore, a significant time by group interaction (*P* = 0.025; effect size = 0.310) was found in the average directional control. The sitting Tai Chi trainees showed a significant improvement (*P* = 0.047) after three months of training while there was no significant change in the control group (*P* = 0.076) over time. The effect sizes ranged from 0.263 to 0.349 which were regarded as from small to moderate effects.

#### 3.2.2. Changes in Sequential Weight Shifting Performance

The total time to complete the sequential weight shifting test showed a significant time by group interaction (*P* = 0.035; effect size = 0.281, Table 2). Paired *t*-tests showed that only the Tai Chi group showed a significant average improvement (*P* = 0.012) after the three months of training. No significant change was found in the control group (*P* = 0.399). Between-group comparisons demonstrated that the difference between the two groups was statistically significant after the intervention (*P* = 0.001).

A significant time by group interaction (*P* = 0.033; effect size = 0.286) was also found in terms of directional control, but there was no significant change in either group after the three-month intervention. The effect sizes ranged from 0.281 to 0.286 which were regarded as from small to moderate effects.

### 3.3. Quality of Life

No significant time by group interaction (*P* > 0.05) was found in any domain of the WHOQOL-BREF results. There was no significant difference between the two groups before or after the intervention (*P* > 0.05).

## 4. Discussion

### 4.1. Handgrip Strength

The experimental group showed improved grip strength in both hands. To the extent that handgrip strength reflects overall body strength in older population [20], the findings may indicate a general improvement in average muscle strength among the sitting Tai Chi trainees. Similar findings have been reported by Jones' group [31]. In that study, fifty-one participants inexperienced in Tai Chi learned 119 forms of Cheng's style Tai Chi in a community center for 1.5 hours, 3 times per week for 12 weeks. Handgrip strength improved significantly after the 3 months of training (average increase of 2.6%). The 12-form Tai Chi used in this study is much simpler and easier to learn; yet it also produced a significant improvement in average handgrip strength (average increase of 12.3%).

The key difference may be that Jones' participants were not wheelchair bound. Previous studies have shown that persons with high level SCI compensate their loss of postural muscle function from the erector spinae through increased use of nonpostural muscles such as the latissimus dorsi, upper trapezius, pectoralis major, and serratus anterior [32–35]. If improved handgrip strength reflects better general muscle strength, that may lead to improved wheelchair maneuvering. However, further research is warranted.

### 4.2. Sitting Balance Control

#### 4.2.1. Limits of Stability

This has been the first study to investigate the effects of sitting Tai Chi on the sitting balance control of community-dwelling people with SCI. The sitting Tai Chi practitioners showed improvements in the limits of stability test and were able to achieve significantly better reaction times, maximum excursions, and directional control. The Tai Chi practitioners showed better dynamic balance control in self-initiated shifting of the COM to different spatial targets within their base of support. Reaction time in initiating movement depends on both neuromuscular control and cognitive factors [36]. The improved muscle strength found in this study and better balance control stabilize the body in advance of potentially destabilizing movements [14], which might explain why the Tai Chi practitioners had shorter reaction times in the voluntary leaning of their body to the eight target positions. The Tai Chi group also showed improved COP distance travelled after the intervention while no significant change was found in the control group. The kinematic characteristics of the sitting Tai Chi forms provide comparatively large anteroposterior and mediolateral COP displacements [18] which may explain these results. However, the baseline values of the maximum excursion between the two groups were significantly different. Though they were treated as covariate in the statistical analysis, the finding should be interpreted with caution.

A previous Tai Chi study has shown that 4-week intensive training (1.5 hr/session, 6 times per week for 24 sessions) significantly improves directional control in the limits of stability test for community-dwelling elderly who are standing. However, those older participants did not improve their average reaction time or maximum excursion [37]. The investigators in that study explained that Tai Chi movements require the practitioners to perform the forms in a smooth and coordinated manner, thus enhancing their directional control. Since the elderly participants were healthy and their initial maximum excursion levels were already considered high, the short-term intensive Tai Chi practice could not further enhance their average maximum excursion. In this present study the participants lived in the community but felt that they lacked exercise after being discharged from the hospital. Practicing sitting Tai Chi may provide an alternative means of exercise which should be effective in improving their balance control.

#### 4.2.2. Sequential Weight Shifting Test

The sequential weight shifting assessment required the participants to shift their weight as quickly and accurately as possible. It demanded acceleration and deceleration of the trunk along both orthogonal and diagonal trajectories which are important in the ADL of those with SCI. Trunk angular displacements and velocity control are key elements in any transfer [38]. A group led by Forslund investigated the coordination of body movement during transfer from a table to a wheelchair among 13 people with SCI. They started with a weight shift from the buttocks to the hands by trunk flexion and rotation towards the trailing side [39]. Sitting Tai Chi involves sequential shifting with reciprocal arm movements and coordinated trunk flexion and rotation. In Tai Chi practice, one of the principles for able bodied individuals is to lead the movement with the “waist” [40]. Also, the Tai Chi forms involve both orthogonal and diagonal movements [17], which may explain the improvements in sequential weight shifting among the experimental group.

Generally, studies investigating exercises to improve the sitting balance control of community-dwelling people with SCI are scarce. Kayak training has been investigated in a few studies [41, 42]. Grigorenko and his colleagues [41] conducted an 8-week open-sea kayak training program with 12 individuals with SCI and 12 able bodied subjects. The results showed a relatively small treatment effect on balance in the sagittal plane during quiet sitting test. In the later study led by Bjerkefors et al. [42], 10 subjects with thoracic SCI were given 10 weeks of kayak arm ergometer training and the investigators found less body sway in rotation and less linear displacement of the trunk during perturbations. These studies only demonstrated improved static posture control, not better dynamic balance control. In any case, neither open sea kayaks nor kayak arm ergometers are easy to access for SCI survivors in the community.

### 4.3. Quality of Life

There was no significant change in the average scores in any of the four QOL domains in either group. The question may thus arise as why there were improvements observed in handgrip strength and dynamic sitting balance control in the Tai Chi group, but no change was reflected in the physical domain by the WHOQOL-BREF questionnaire. One possible reason is that all the participants were at the chronic stage and their social and environment domains had reached a steady state. It may take more time for improved strength and balance control to be reflected in improvements in daily life. In this connection, Hicks and his colleagues found that the quality of life of individuals with SCI was improved only after 9 months of twice-weekly exercise training but not after three or six months [43]. Some participants in this study did, however, mention that they had better access to their living environment after the Tai Chi training. For example, one participant reported that he could lean further to get a magazine from the newspaper stand and another Tai Chi participant commented that he had better mobility while accessing the mass transit railway.

### 4.4. Limitations of Study

The present study had several limitations. The participants in this study were heterogeneous in terms of age and gender, injury level, and time since injury. The significant changes in the handgrip strength and sitting balance control in the sitting Tai Chi participants should therefore be generalized to others with SCI only with caution. The small sample size is a common problem among studies on SCI, but that too limits generalizability. The postassessment was conducted immediately after the intervention, so any long-term effect of Tai Chi training on sitting balance control and ADL was not ascertained. In this pilot study, the overall statistical result of each domain, namely, handgrip strength, sitting balance control, and QOL, was reported first and followed by each outcome measure. However, the *P* values in the subsequent multiple univariate analyses were not adjusted which might increase the probability of committing a type I error. Therefore, the findings should be interpreted with caution. Moreover, the participants were not randomly allocated to the Tai Chi or control groups, so experimental bias might have occurred. Measurement bias might also be present because our wooden force plate is comprised of load cells which did not measure the horizontal force component directly. This may result in some inaccuracy of the COP measurement. Therefore, further study with a randomized clinical trial design and using precise measuring instrument is needed.

## 5. Conclusions

Twelve weeks of sitting Tai Chi training could improve the dynamic sitting balance performance and handgrip strength, but not QOL, of the community-dwelling SCI survivors. Results of this pilot study substantiate the feasibility of the sitting Tai Chi training for them. However, a larger scale of randomized controlled trial is required to verify the effectiveness of sitting Tai Chi training on the muscle strength, balance control, and QOL of people with SCI. If the findings are positive, clinicians may recommend sitting Tai Chi as a health-maintenance exercise to people with SCI. This particular group of survivors may continue to practice the sitting Tai Chi exercise even after being discharged from hospital and in the community.

## Figures and Tables

**Table 1:** Characteristics of the participants.

	Control group (*n* = 8)	Sitting Tai Chi group (*n* = 11)	*P* value
Age, years	46.2 ± 11.8	49.1 ± 10.3	0.761
Gender (male/female), *n*	7/1	4/7	0.062
Sitting height, cm	83.7 ± 5.5	75.5 ± 11.1	0.122
Weight, kg	65.4 ± 14.6	60.4 ± 24.3	0.064
Injury level (high/low), *n*	5/3	7/4	0.968
C6-T1	3	6	
T2-T7	2	1	
T8-L1	1	4	
ASIA			0.236
B	6	5
C	0	3
D	2	3	
Time since injury, years	17.3 ± 7.8	14.7 ± 13.7	0.258

*Note*. Values are mean ± SD or *P* values.

ASIA: American Spinal Injury Association Impairment Scale.

**Table 2:** Comparison of outcome measurements between and within groups.

Measurements	Control group (*n* = 8)		Sitting Tai Chi group (*n* = 11)		*P* value			
	Pretest	Posttest	Pretest	Posttest	Pretest (between-group comparison)	Posttest (between-group comparison)	Group × time effect	Effect size of interaction
Handgrip strength								
Right hand (kg)	32.8 ± 17.1	31.5 ± 14.8	21.4 ± 13.4	24.8 ± 15.2^f^	0.354	0.807	0.019^a^	0.285
Left hand (kg)	31.6 ± 15.4	32.4 ± 14.8	21.2 ± 11.8	23.8 ± 12.4^e^	0.473	0.924	0.318	0.059
Limits of stability test								
Reaction time (sec)	0.72 ± 0.27	0.83 ± 0.42	1.03 ± 0.33	0.76 ± 0.16^e^	0.580	0.144	0.042^a^	0.263
Maximum excursion (mm)	61.9 ± 53.6	60.6 ± 50.1	35.4 ± 19.8	44.4 ± 21.7^f^	0.005^d^	0.015^c^	0.016^a^	0.349
Directional control (%)	0.68 ± 0.1	0.65 ± 0.12	0.70 ± 0.11	0.77 ± 0.09^e^	0.725	0.555	0.025^a^	0.310
Sequential weight shifting test								
Total time (sec)	50.8 ± 16.6	59.2 ± 27.5	55.0 ± 8.7	41.5 ± 7.8^e^	0.190	0.001^d^	0.035^a^	0.281
Directional control (%)	0.64 ± 0.09	0.61 ± 0.09	0.60 ± 0.07	0.64 ± 0.04	0.699	0.027^c^	0.033^a^	0.286
Quality of life								
Physical	10.6 ± 1.7	10.6 ± 1.8	11.4 ± 1.3	10.8 ± 2.1	0.331	0.490	0.534	0.023
Psychological	12.5 ± 1.9	12.2 ± 1.8	11.3 ± 1.4	11.8 ±2.2	0.308	0.741	0.171	0.107
Social	12.3 ± 2.6	12.8 ± 2.4	13.9 ± 2.5	14.1 ± 2.5	0.584	0.924	0.736	0.007
Environmental	13.3 ± 2.3	12.7 ± 2.6	11.9 ± 2.4	13.4 ± 4.9	0.697	0.373	0.196	0.096

*Note*. Values are mean ± SD or *P* values.

Group by time interaction.

^a^A difference significant at the *P* < 0.05 confidence level.

^c^A difference significant at the *P* < 0.05 confidence level.

^d^A difference significant at the *P* < 0.01 level of confidence.

Within group (time effect).

^e^A difference significant at the *P* < 0.05 level when compared with pretest values.

^f^A difference significant at the *P* < 0.01 level when compared with pretest values.
